# Potential tumor-specific antigens and immune landscapes identification for mRNA vaccine in thyroid cancer

**DOI:** 10.3389/fonc.2024.1480028

**Published:** 2024-09-30

**Authors:** Xiaoning Wang, Guixin Wang, Qiaoqiao Xu, Yingxi Li, Wenbin Song, Zhaoyi Liu, Yao Tian, Li Wang, Ke Zhao, Yizeng Wang

**Affiliations:** ^1^ Department of General Surgery, Tianjin Medical University General Hospital, Tianjin Key Laboratory of Precise Vascular Reconstruction and Organ Function Repair, Tianjin General Surgery Institute, Tianjin, China; ^2^ The First Department of Breast Cancer, Tianjin Medical University Cancer Institute and Hospital, National Clinical Research Center for Cancer, Tianjin, China; ^3^ Immunology Department, Key Laboratory of Immune Microenvironment and Disease (Ministry of Education), Tianjin Medical University, Tianjin, China; ^4^ National Clinical Research Center of Cancer, Tianjin Key Laboratory of Cancer Prevention and Therapy, Tianjin’s Clinical Research Center of Cancer, Tianjin Medical University Cancer Institute and Hospital, Tianjin Medical University, Tianjin, China

**Keywords:** thyroid cancer, mRNA vaccine, immune landscape, tumor immune microenvironment, TK1, immunotype

## Abstract

**Background:**

Tumor mRNA vaccines have been identified as a promising technology for cancer therapy in multiple cancer types, while their efficacy in thyroid cancer (THCA) is unclear. Immunotyping is strongly associated with the immune microenvironment and immune status in cancer, thus it is important in vaccination and therapeutic response. This study is to identify potential valuable antigens and novel immune subtypes of THCA for immune landscape construction, thus screening patients suitable for mRNA vaccination.

**Methods:**

The clinical information and gene expression files of 568 THCA cases were obtained from the TCGA dataset. The DNA copy number variation and the somatic mutation of THCA were visualized by the cBioPortal database. TIMER was used to investigate the immune infiltrating correlation with candidate antigens. Consensus clustering analysis was conducted to cluster data using the ConsensusClusterPlus package. The immune landscapes of THCA patients were visualized using the Monocle package. The critical hub genes for THCA mRNA vaccines were identified by WGCNA package. To validate, the immunohistochemistry and real-time quantitative PCR (RT-qPCR) were performed to detect the expression level of potential antigen for mRNA vaccine in tissue and cell lines in THCA.

**Results:**

Thymidine kinase 1 (*TK1*) was identified as a potential biomarker of mRNA vaccine against THCA. It was confirmed to be significantly upregulated in THCA tissues and cells lines. Moreover, three novel immune subtypes of THCA were obtained based on the expression consistency of immune-associated genes. The S2 subtype was characterized as an immunological “cold” phenotype with a high expression of immunogenic cell death modulators. S1 and S3 subtypes were immunological “hot” phenotypes with immune checkpoints upregulation. Further, the immune landscape of THCA patients was visualized and ten hub genes for mRNA vaccines were identified.

**Conclusion:**

*TK1* was a tumor-specific antigen of mRNA vaccines. The patients belonging to the S2 subtype (“cold” tumor) were suitable for mRNA vaccine therapy in THCA. Notably, ten hub genes were conducted as potential biomarkers for identifying suitable patients for mRNA vaccination. These findings provided novel insights into mRNA vaccine development against THCA.

## Introduction

Thyroid cancer (THCA) is the most frequently diagnosed endocrine malignancy worldwide, and its incidence has recently increased (1). Thyroid cancers are subdivided into four subtypes according to their pathological features, including anaplastic thyroid cancer (ATC), follicular thyroid cancer (FTC), medullary thyroid cancer (MTC), and papillary thyroid cancer (PTC) (2). The well-differentiated subtypes of THCA, namely FTC and PTC, tend to have a better prognosis, whereas the poor/undifferentiated-differentiated subtype of THCA is associated with a poor prognosis (3, 4). Studies have demonstrated several novel therapies for THCA patients, including immunotherapy, although their efficacy still needs further verification (5, 6). Thus, identifying new biomarkers and investigating emerging therapy strategies are important.

Therapeutic cancer vaccines have a strong biological and preclinical theoretical basis and have undergone continuous development (7). Many clinical trials utilized vaccine designs or target antigens to induce an immune response (8). Cancer vaccines are divided into four main categories, namely viral vector-based vaccines, tumor or immune cell-based vaccines, nucleic acid-based vaccines and peptide-based vaccines (9). Nucleic acid-based vaccines, formulated from DNA or RNA, are an emerging vaccine platform in cancer therapy that can induce both cell and humoral mediated immune responses (10). RNA vaccines exhibit higher rates and magnitudes of protein expression than DNA vaccines (11). Recently, mRNA-based immune therapies have undergone clinical trials in the treatment of multiple diseases (12, 13), as well as being a key weapon in controlling the spread of the coronavirus disease 2019 (COVID-19) (14, 15). Nevertheless, not all patients can benefit from mRNA vaccines, which can turn a cold tumor into hot by promoting T cell response (16).

The development of mRNA vaccines has achieved major improvements in various vaccines. Huang et al. developed potent antigens for developing an mRNA vaccine against pancreatic adenocarcinoma (17). Several candidate tumor-specific antigens in bladder cancer have been identified by Wang et al. (18). However, THCA-mRNA vaccine development has not seen the same level of progress due to the heterogeneity (immune response, clinical stage, metastasis) of the patients making the filter of tumor-specific antigens still challenging. Thus, further investigation is necessary to promote the progress of mRNA vaccines in THCA treatment.

In this study, we identified *TK1* as a potential tumor associated antigen and constructed the immune landscapes of thyroid cancer. Furthermore, we identified 10 hub genes as potential biomarkers for screening the specific population for mRNA vaccination. Our work was valuable for identifying suitable patients for mRNA vaccines, which might provide emerging immune therapy strategies for THCA patients.

## Materials and methods

### Cell culture

The human thyroid carcinoma cell lines B-CPAP, TPC-1, K-1, T238, THJ-16T, OCUT-2, SW1736 cell lines and normal thyroid follicular epithelial cells Nthy-ori 3-1 were obtained from the American Tissue Culture Collection (ATCC, Manassas, VA, USA). Normal thyroid follicular epithelial cells and thyroid carcinoma cells were cultured in F12K and RPMI 1640 medium respectively, supplemented with 10% fetal bovine serum (FBS) and 1% penicillin/streptomycin in a 5% CO_2_ and humidified atmosphere at 37°C.

### Immunohistochemistry

For patient samples, written informed consent was obtained from each patient and the study was approved by the hospital ethics committee (Tianjin Medical University Cancer Institute and Hospital). Tumor samples and the paired normal samples were patients suffering from THCA in our hospital. Tissue sections were deparaffinized, rehydrated, and permeated using Triton X 100 (T8200, Solarbio, China) and followed by antigen retrieval using EDTA Antigen Retrieval solution (c1034, Solarbio, China). The sections were incubated with Anti-TK1 antibody (15691-1-AP, Proteintech, China) at 4°C overnight followed by a biotinylated secondary antibody (diluted at 1:200) at RT for 60 min. Then, the sections were stained with DAB staining solution (AR1022, BOSTER Biological Technology, China). To quantitatively evaluate, the extent of staining was scored as the following criteria: (a) percentage of stained cells: 0 (0%), 1 (1%–25%), 2 (26%–75%), 3 (51%–75%) and 4 (> 75%); and (b) staining intensity: 0 (negative staining), 1 (low staining), 2 (medium staining), and 3 (high staining). The final scores were calculated by multiplying the scores of intensities with that of extent.

### Real-time quantitative PCR

The mRNA from thyroid cancer tissues and cells were extracted by using RNA extraction kit (Spark Jade, China), and reversed by using Uni All-in-One First-Strand cDNA Synthesis SuperMix for qPCR (One-Step gDNA Removal) (Transgene, China). The primers used for TK1 were selected as: 5’-GTTTTTCCCTGACATCGTGGA-3’; 5’- CGAGCCTCTTGGTATAGGCG-3’. The RT-qPCR was performed according to the manufactures’ instructions of One-Step RT-PCR SuperMix (Transgene, China).

### Data extraction and preprocessing

The clinical data and gene expression profiles of 568 thyroid cancer patients were downloaded from The Cancer Genome Atlas (TCGA, https://www.cancer.gov/tcga) and normalized. The inclusion criteria are the patients suffering from thyroid cancer. Simultaneously, patients who don’t have complete disease-free survival is excluded in our study. The immune-associated genes were collected from IMMPORT dataset (https://immport.niaid.nih.gov/home).

To process data, 60483 probes was utilized to map TCGA-THCA datasets. The gene expression pattern of THCA was normalized for the following analyses. The samples without complete clinical information were excluded, leaving 488 samples included for the following analysis.

### GEPIA analysis and survival analysis

Patient survival and differential gene expression data were analyzed using GEPIA2 (20). To perform differential analysis, the ANOVA method was conducted, and over-expressed genes were thought as logFC >1 and *P*-value<0.05.

Kaplan-Meier curve was utilized to estimate the correlation between candidates and clinical outcomes. The median value was set for group cutoff. Values with *P*<0.05 were considered statistically significant.

### cBioPortal analysis

The cBioPortal (http://www.cbioportal.org) was used to integrate the single nucleotide variation (SNV) and DNA copy number variation (CNV) of TCGA database (19). The gene alternations of THCA were compared.

### TIMER analysis

TIMER database (https://cistrome.shinyapps.io/timer/) was conducted to analyze and visualize the correlation between immune cells (dendritic cells, macrophages, and B cells) and the candidate target genes (21). Values with *P*<0.05 were considered statistically significant. Spearman’s correlation analysis was performed for purity adjustment.

### Construction of novel immune subtypes

The total number of genes was identified through univariate Cox hazard analysis, among which 105 significant immune-related genes were filtered. A consistency matrix was constructed using the ConsensusClusterPlus package to discover the immune subtypes. One hundred bootstraps were performed using the PAM (partitioning around medoids) algorithm, which involved 80% of the patients in the discovery cohort. Cluster sets varied from 2 to 6. The clinical details (age, gender, disease-free status, tumor stage, pathological T stage) of the immune subtypes were further analyzed.

### Prognostic evaluation of immune subtypes

Kaplan-Meier curve was utilized to perform a survival analysis for distinct immune subtypes of patients. The enrichment scores of immune-associated signatures and immune infiltrating cells were calculated for each patient using single-sample GSEA (ssGSEA). The significance between subtypes was tested via the Kruskal-Wallis test. Values with p<0.05 were considered statistically significant.

### Construction of immune landscapes

The monocle package was conducted to reduce dimensionality for individual THCA patients according to gene expression profiles. The DDRTree method was used for reducing dimension and all patients were ordered. Pearson correlation analysis was used to analyze the correlation between immune cells and immune landscapes. Values with p<0.05 were considered statistically significant.

### Gene co-expression network

The co-expression modules of the immune-associated genes were constructed by WGCNA (weighted correlation network analysis) package (22). The prognostic value of different gene modules was calculated by Cox regression analysis. The clusterProfiler package was utilized to conduct kyoto encyclopedia of genes and genomes (KEGG) and gene ontology (GO) analysis. The correlation between the component 1 of immune landscape and the brown module was evaluated via Pearson correlation analysis. Finally, protein-protein interaction analysis was performed to find the core genes of the brown module through Metascape (https://metascape.org/gp/index.html#/main/step1).

## Results

### Identifying the candidate antigens in THCA

**Figure 1:**
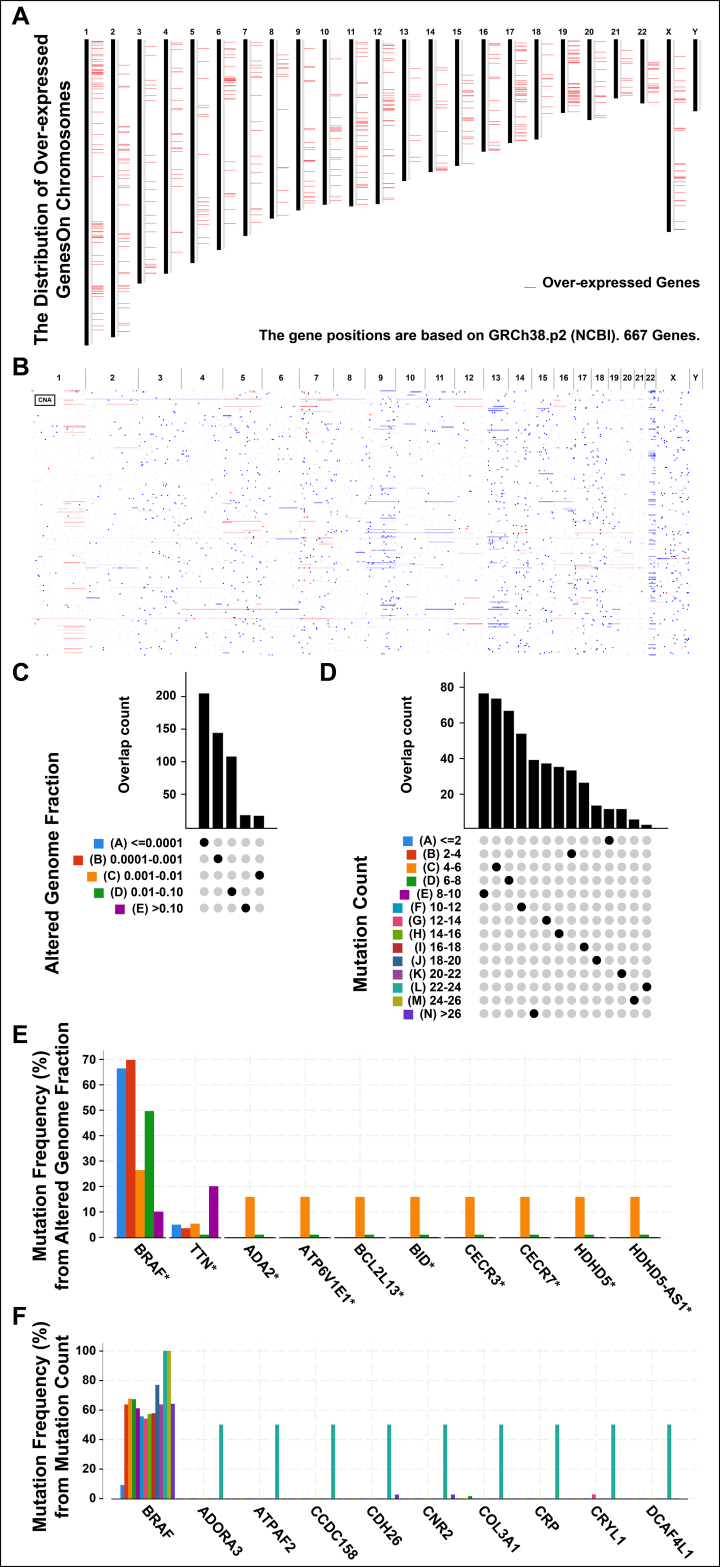
Identifying the candidate antigens in thyroid cancer (THCA). **(A)** Chromosomal distribution of up-regulated genes in THCA. **(B)** The aberrant copy number genes, amplified (red) and deleted (blue) were shown in THCA. **(C)** The overlap count of samples in altered genome fraction. **(A)**, <=0.0001; **(B)**, 0.0001-0.001; **(C)**, 0.001-0.01; **(D)**, 0.01-0.10; **(E)**, >0.10. **(D)** The overlap count of samples in mutation count. **(E)** The genes with the highest frequency in the altered genome alteration group. **(F)** The genes with the highest frequency in the mutation count group. *P < 0.05, **P < 0.01, ***P <0.001, and ‘ns’ represents insignificant.

Tumor antigens are usually derived from over-expressed genes, amplified genes, deleted genes and mutated genes. Some of these antigens have been used in the development of tumor vaccines based on their identification (23, 24). Firstly, to screen for the tumor-specific antigens of THCA, 667 over-expressed genes were detected by GEPIA2, which may encode potential tumor-associated antigens (**Figure 1A**). Secondly, in **Figure 1B**, the number of copies of aberrated genes, which were amplified or deleted, was further analyzed. Lastly, the altered genome fraction (**Figure 1C**) and mutation count (**Figures 1D**) were grouped in TCGA-THCA dataset. The results showed the patient distributions in different mutation counts and altered genome fraction group. Interestingly, in the top ten mutated genes, B-Raf Proto-Oncogene, Serine/Threonine Kinase (*BRAF*) was revealed to be the most frequently mutated gene in terms of both altered genome fraction (**Figures 1E**) and mutation count (**Figure 1F**). In addition to *BRAF* and Titin (*TTN*), Adenosine Deaminase 2 (*ADA2*), ATPase H+ Transporting V1 Subunit E1 (*ATP6V1E1*), BCL2 Like 13 (*BCL2L13*), BH3 Interacting Domain Death Agonist (*BID*), Cat Eye Syndrome Chromosome Region, Candidate 3 (*CECR3*), Cat Eye Syndrome Chromosome Region, Candidate 7 (*CECR7*), Haloacid Dehalogenase Like Hydrolase Domain Containing 5 (*HDHD5*), and *HDHD5* Antisense RNA 1 (*HDHD5-AS1*), were exhibited the same mutation frequencies from altered genome fraction (**Figure 1E**). Furthermore, Adenosine A3 Receptor (*ADORA3*), ATP Synthase Mitochondrial F1 Complex Assembly Factor 2 (*ATPAF2*), Coiled-Coil Domain Containing 158 (*CCDC158*), Cadherin 26 (*CDH26*), Cannabinoid Receptor 2 (*CNR2*), Collagen Type III Alpha 1 Chain (*COL3A1*), C-Reactive Protein (*CRP*), Crystallin Lambda 1 (*CRYL1*), *DDB1* and *CUL4* Associated Factor 4 Like 1 (*DCAF4L1*) were exhibited from mutation count (**Figure 1F**). Overall, 5152 frequently mutated genes, 667 over-expressed genes and 4218 amplified genes were identified, which might serve as tumor-specific antigen candidates in THCA.

### Identifying the prognosis-related potential antigens in THCA

**Figure 2:**
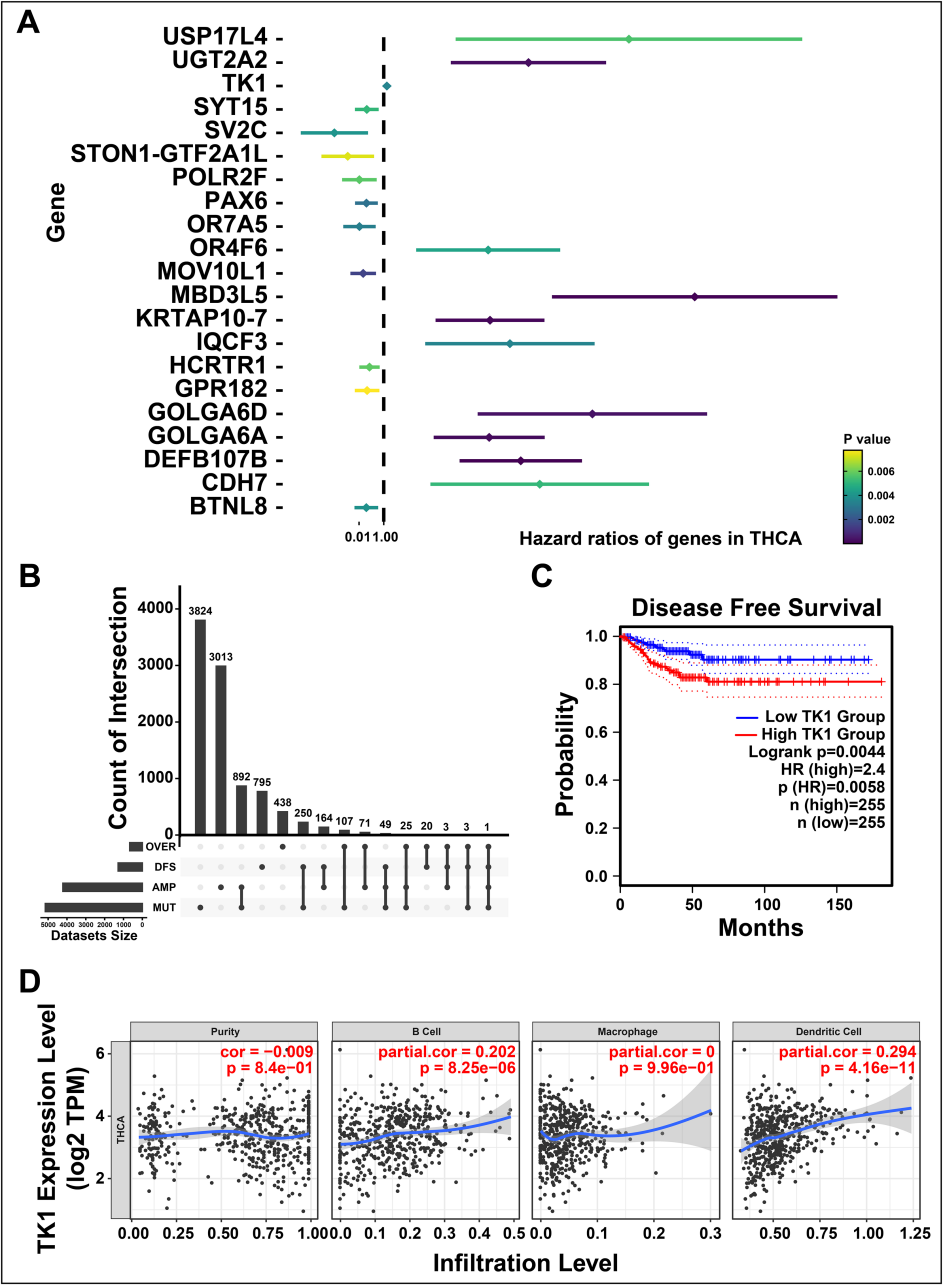
Identifying the prognosis-related potential antigens in thyroid cancer (THCA). **(A)** Forest maps of single factor survival analysis of genes in THCA. **(B)** The upset diagram indicates the number of gene intersections in distinct groups. **(C)** Survival analysis of thymidine kinase 1 (TK1) in THCA performed by Kaplan-Meier (KM) plotter. **(D)** Correlation between TK1 expression with the purity of infiltrating cells and amount of B cells, macrophages, and dendritic cells. ‘ns’ represents insignificant.

To further investigate the prognosis-related tumor antigens, we performed a univariate Cox hazard analysis to illustrate the prognostic value of candidate gene (**Figure 2A**). Notably, *TK1* was identified as a potential tumor-associated antigen in THCA through four gene sets, including over-expressed, DFS-related (disease free survival-related), mutational, and amplified genes (**Figure 2B**). For validation, a Kaplan-Meier survival curve was constructed and found upregulated *TK1* was associated with the worse clinical outcomes in patients of THCA (**Figure 2C**). We further investigated the correlation between *TK1* and immune cells and found that *TK1* exhibited a close association with the increased tumor infiltration of antigen-presenting cells, including dendritic cells (P < 8.25e-06) and B cells (P < 4.16e-11, **Figure 2D**). These results indicated that *TK1* considered as a prognosis-related potential antigen for developing mRNA vaccine against THCA. Then, the antigen was presented by antigen-presenting cells to T cells and simultaneously recognized by B cells to trigger an immune response. To validate, the *TK1* expression levels in thyroid cancer were detected by immunohistochemistry and RT-qPCR. The results exhibited that *TK1* was significantly upregulated in both THCA tissues (**Supplementary Figures S1A, C**) and THCA cells lines (**Supplementary Figure S1B**) compared with normal.

### Identifying the novel immune subtypes of THCA

**Figure 3:**
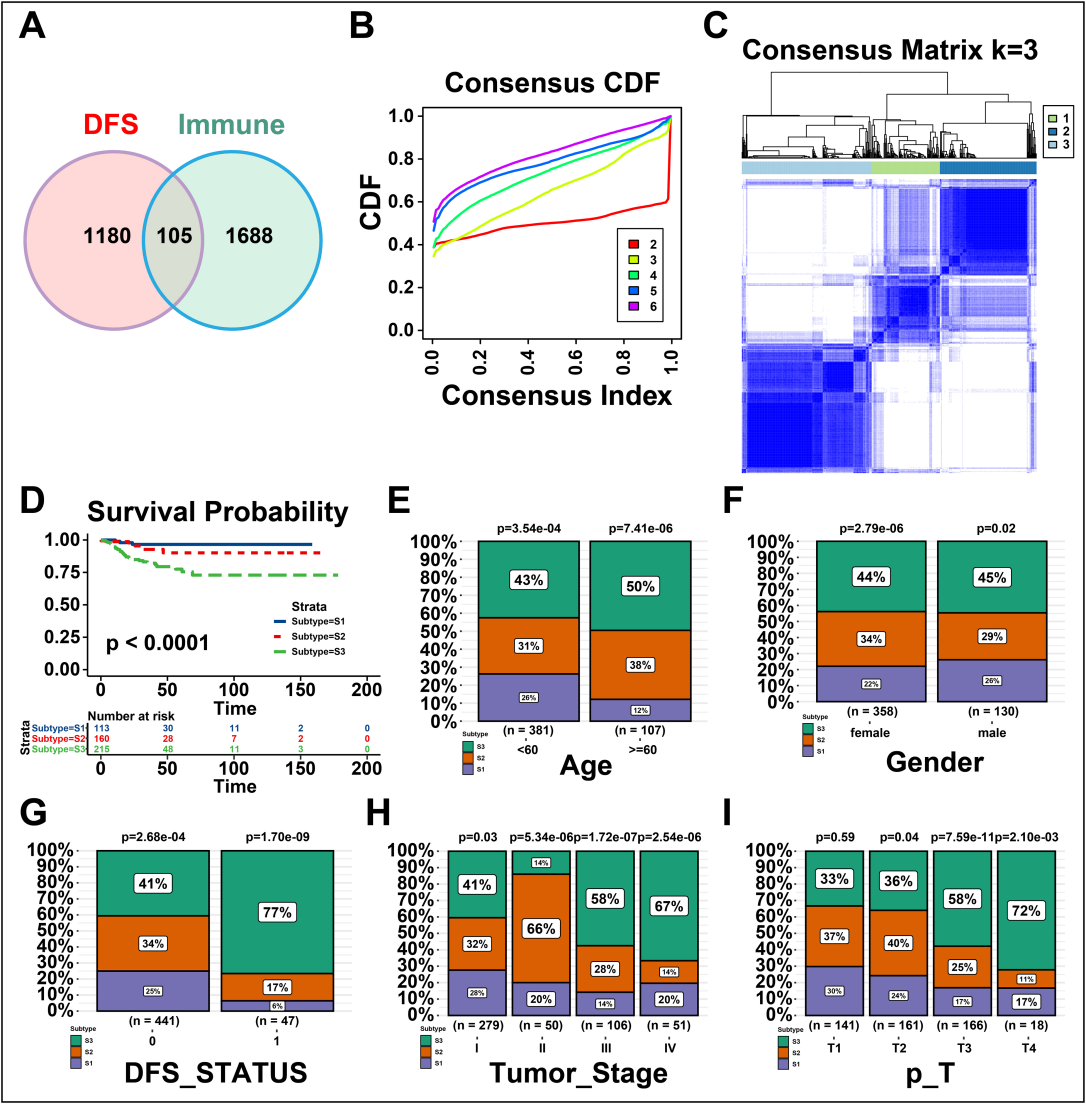
Identifying the novel immune subtypes of thyroid cancer (THCA). **(A)** Venn diagram screening genes from significant disease-free survival (DFS) and immune infiltration in THCA. **(B)** Cumulative distribution function curve of immune-related genes in THCA cohort. **(C)** Sample clustering heatmap. **(D)** Survival analysis of three immune subtypes. **(E-I)** Distribution of S1-S3 across THCA **(E)** age, **(F)** gender, **(G)** DFS status, and **(H, I)** tumor stages. ‘ns’ represents insignificant.

The immune subtypes can exhibit immune state and microenvironment of tumors, thereby it may be essential to identify novel immune subtypes of THCA for mRNA vaccine. To identify immune subtypes of THCA, 105 genes were obtained from the intersection of 1688 immune-related genes and 1180 prognosis-related genes of THCA (**Figure 3A**). These 105 genes were utilized to construct consensus clustering with PAM algorithm. According to the consensus curve, the genes were stably grouped when k = 3 (**Figure 3B**). Then, we finally obtained three immune subtypes of THCA which were designated as S1, S2, and S3 (**Figure 3C**). S1 was related to a better prognosis, while S2 and S3 had a poor prognosis (**Figure 3D**). Further, the clinical differences among subtypes were exhibited in **Figures 3E–I**. The clinical characteristics including higher age (>=60), DFS status (disease progressed), higher tumor stage and T stage (advanced) were closely associated with S3, while gender showed no apparent difference in S3. Taken together, three novel immune subtypes correlated with the prognosis of THCA patients are identified. And immune subtypes could distinguish the clinical characteristics of THCA patients.

### Correlation between cellular and molecular characteristics with immune sub-types

**Figure 4:**
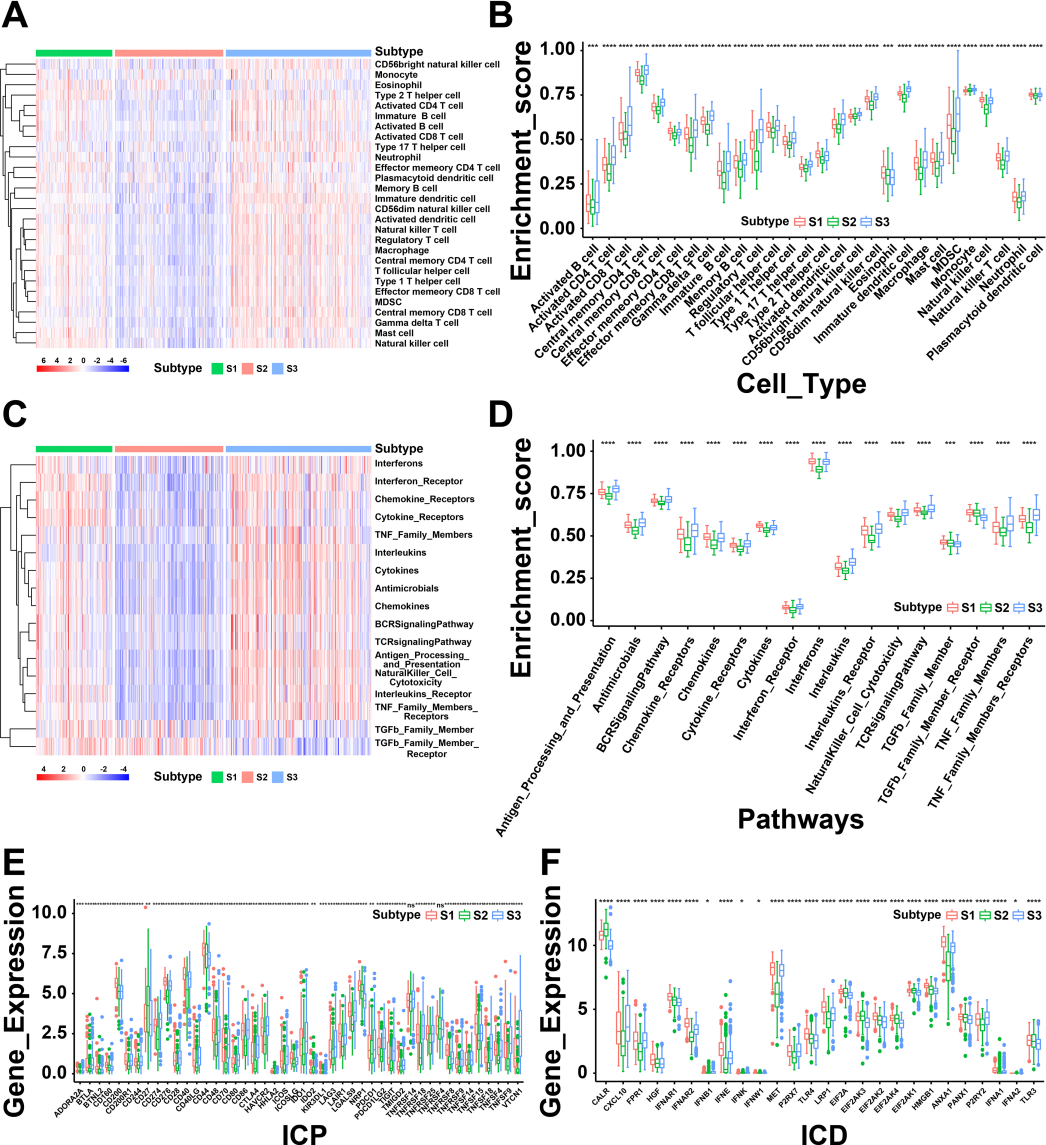
A correlation between cellular and molecular characteristics with three immune subtypes of thyroid cancer (THCA). **(A)** Heatmap of differential enrichment scores of 28 immune cell signatures among THCA immune subtypes. **(B)** Boxplot of differential enrichment scores of 28 immune cell signatures among THCA immune subtypes. **(C)** Heatmap of differential enrichment scores of immune-related pathways among three immune subtypes. **(D)** Boxplot of differential enrichment scores of immune-related pathways among three immune subtypes. **(E)** Differential expression of immune checkpoint (ICP) genes among THCA immune subtypes. **(F)** Differential expression of immunogenic cell death (ICD) modulator genes among THCA immune subtypes. *P < 0.05, **P < 0.01, ***P <0.001, **** P <0.0001 and ‘ns’ represents insignificant.

The immune status of tumor is strongly associated with the immune response. Thus, we next investigated the immune cell proportions among three subtypes by calculating the enrichment score of 28 tumor-infiltrating lymphocyte markers using ssGSEA. As shown in **Figure 4A**, the results displayed that S1 and S3 had more immune infiltrating cells, while S2 showed a lower immune cell infiltration. To be specific, the score of enriched immune cells including activated B cells, activated CD4 T cells, activated CD8 T cells, macrophages and activated dendritic cells showed lower in S2 than S1 and S3 (**Figure 4B**). Furthermore, the immune-related pathways were analyzed and found that genes of chemokine receptors, interferon receptors, and interferons were down-regulated in S2 (**Figures 4C, D**). In summary, the results revealed that S1 and S3 are immunological “hot” phenotypes, however, S2 is an immunological “cold” phenotype. It has been confirmed that tumor vaccines could turn the immunological “cold” tumor into “hot” tumor by increasing the number of antigen-specific T cells (25). Therefore, the mRNA vaccine in THCA might be beneficial for the patients of “cold” S2 tumor.

Next, we further analyzed the expression levels of immunogenic cell death (ICD) modulators and immune checkpoints (ICPs) in the three subtypes, which may be valuable biomarkers for mRNA vaccines. Forty-five ICP-related genes were differentially expressed among the immune subtypes. For instance, the expression levels of *BTLA, CD86, CD70, BTNL2, CD200R1, CD28, CD40, CD40LG, CD48, CD80, CD244, CTLA4, IDO1, HAVCR2, CD274, ICOS, ICOSLG, CD276* and many other ICPs were significantly downregulated in S2 than S1 and S3 (**Figure 4E**). The expression levels of ICD-associated genes (*CALR, EIF2A, EIF2AK3, EIF2AK4*, and *TLR3*) were remarkably elevated in S2 (**Figure 4F**). Based above, these findings indicated the immune subtypes could reflect the expression pattern of ICD modulators and ICPs. Furthermore, patients with “cold” S2 tumor were potentially beneficial from mRNA vaccines.

### The immune landscape of THCA

**Figure 5:**
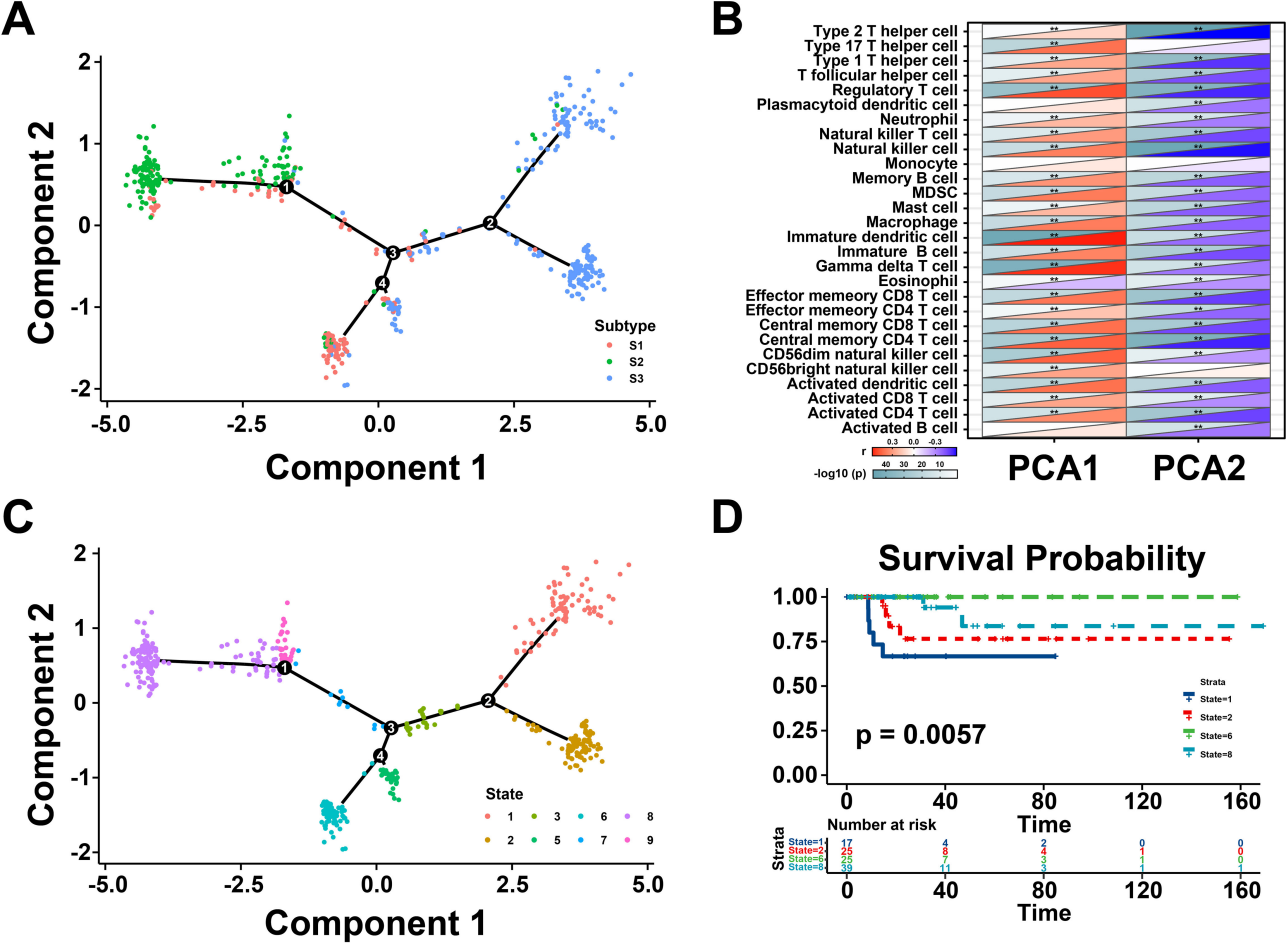
Immune landscape of thyroid cancer (THCA). **(A)** The immune landscape of THCA. The location of individual patients in the immune landscape with the color corresponding to the immune subtypes. **(B)** Correlation between PCA1/2 and immune modules. **(C)** Patients are separated by the immune landscape based on their location. **(D)** Separated patients were associated with different clinical outcomes. **P < 0.01, and ‘ns’ represents insignificant.

Next, the immune landscape of each THCA patient were visualized and calculated based on the immune gene profiles by monocle package (**Figure 5A**). According to the dimensionality reduction of three immune subtypes, S1 and S3 exhibited different distributions compared with S2, which suggest that three immune subtypes could be separated distinctly by immune landscape we constructed. Subsequently, the association between component 1&2 and immune cell infiltration was calculated by Pearson correlation (**Figure 5B**). The X-axis (Component 1) was associated with various tumor-infiltrating lymphocytes, such as regulatory T cell, natural killer cell, immature dendritic cell, and gamma delta T cell. All patients were clustered into different groups to further investigate the prognostic value of the landscape (**Figure 5C**). According to the location of the immune cell populations, groups 1, 2, 6, and 8 were selected for survival analysis, and the results depicted that these patients were distinct from the rest of the distribution (**Figure 5D**). To sum up, these results revealed that the immune landscape was valuable for identifying suitable patients for mRNA vaccines and exhibited a strong correlation with their prognosis.

### Immune gene co-expression modules of THCA

**Figure 6:**
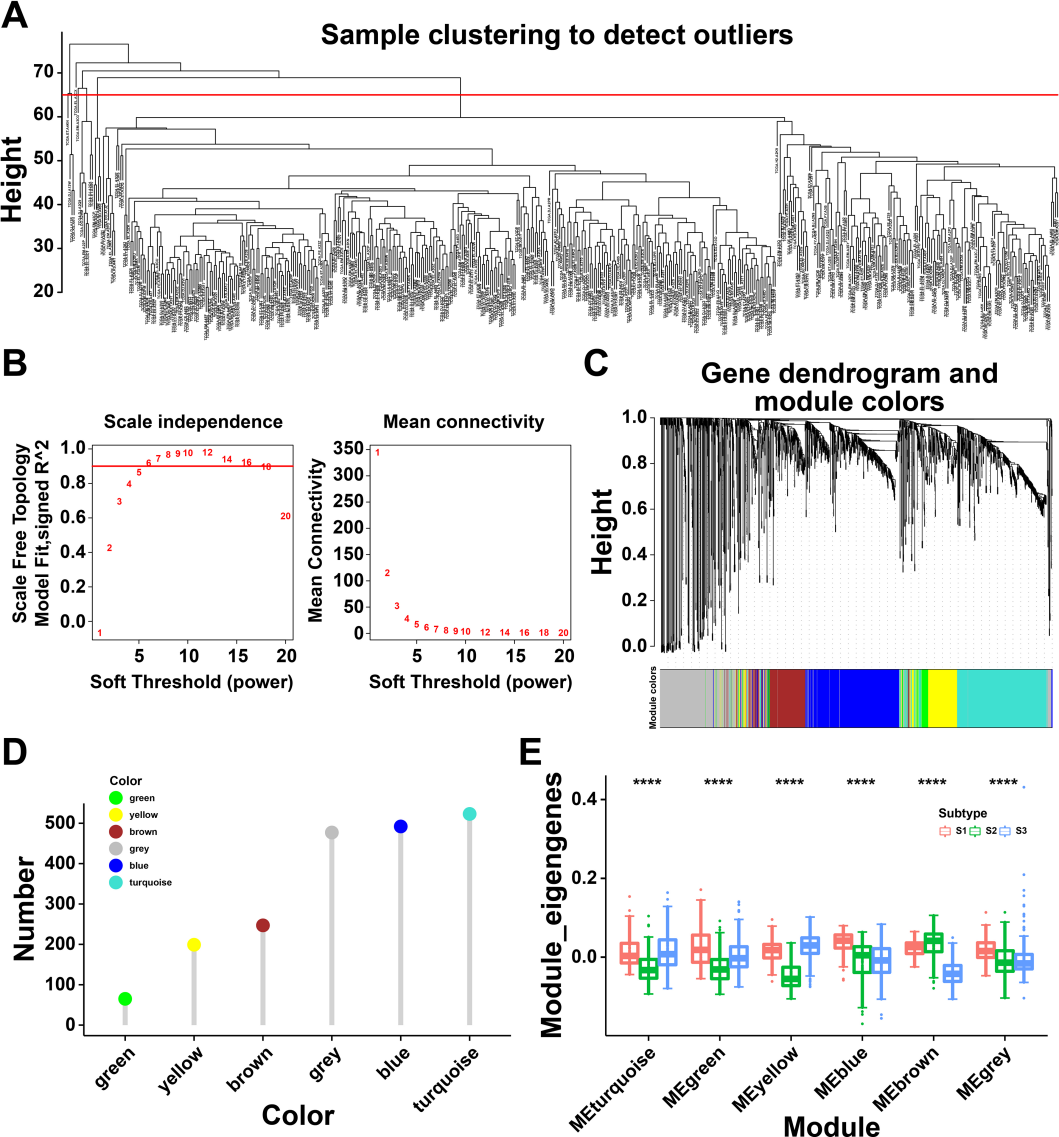
Immune gene co-expression modules of thyroid cancer (THCA). **(A)** Sample clustering. **(B)** Scale-free fit index for various soft-thresholding powers and their mean connectivity. **(C)** Dendrogram of all differentially expressed genes clustered based on a dissimilarity measure (1-TOM). **(D)** Gene numbers in each module. **(E)** Differential distribution of feature vectors of each module in THCA subtypes. **** P<0.0001 and ‘ns’ represents insignificant.

To identify the critical genes related to immune subtypes, WGCNA package was conducted to analyze the immune gene co-expression modules (**Figure 6A**), and 17 outlier samples were excluded. The immune gene co-expression network appeared to be stable when soft threshold was set as 6 for a scale-free network (**Figure 6B**). Then, six gene modules were obtained (**Figure 6C**). The gene numbers of each module are illustrated in **Figure 6D**. We next calculated the eigengenes of three immune subtypes in different modules, demonstrating that the module eigengenes of S2 were notably higher in the brown module but lower in other modules (**Figure 6E**). These findings identified differential gene modules, among which, the eigengenes of S2 were highly expressed in the brown module, indicated the genes of brown module might serve as potential biomarkers for S2 subtype.

### Identifying the hub genes of mRNA vaccine for THCA

**Figure 7:**
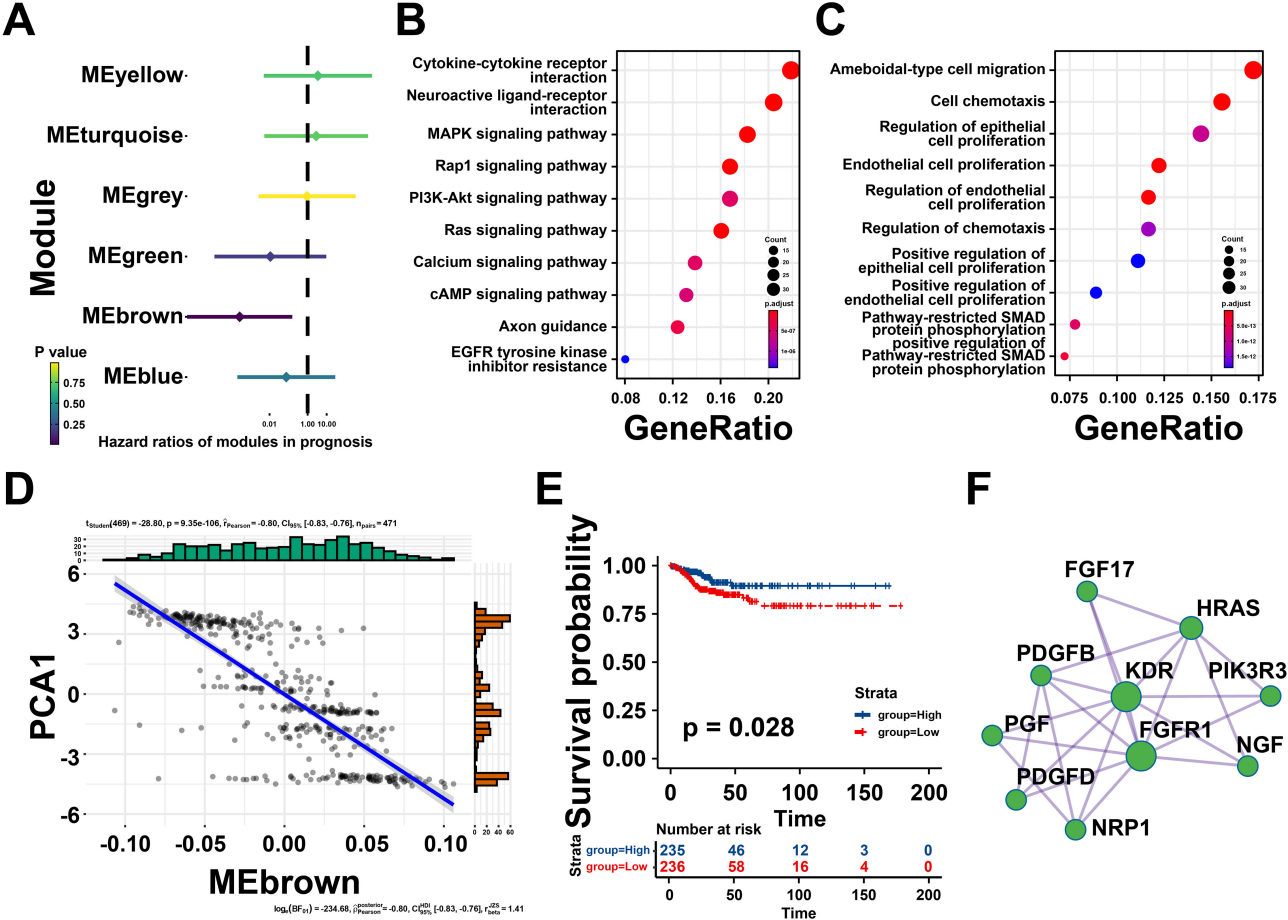
Identifying the hub genes of mRNA vaccine for thyroid cancer (THCA). **(A)** Forest maps of single factor survival analysis of six modules of THCA. **(B)** Bubble plot showing top 10 kyoto encyclopedia of genes and genomes (KEGG) terms in the brown module. **(C)** The top 10 enriched terms in gene ontology (GO) analysis of brown module. **(D)** Correlation between the brown module feature vector and PCA1 in the immune landscape. **(E)** Differential prognosis in the brown module with high and low mean. **(F)** Protein-protein interaction (PPI) network of hub genes identified in the brown module. *P < 0.05, **P < 0.01, ***P <0.001, and ‘ns’ represents insignificant.

Univariate Cox regression analysis was utilized to estimate the prognostic effect of different gene modules, which indicated that the brown module was closely associated with thyroid cancer prognosis (**Figure 7A**). Then, the genes extracted from the brown module were subjected to KEGG analysis and were highly enriched in neuroactive ligand-receptor interaction, MAPK signaling pathway, and cytokine-cytokine receptor interaction (**Figure 7B**). Meanwhile, GO analysis suggested that the brown module genes were mainly involved in ameboid-type cell migration, cell chemotaxis and regulation of epithelial cell proliferation (**Figure 7C**). Notably, the eigengenes of brown modules was negatively related to component 1 of immune landscape, suggesting that the genes in brown module could be predictors for patients unsuitable for mRNA vaccines (**Figure 7D**). Besides, patients with high brown module gene expression levels exhibited a better prognosis (**Figure 7E**). Taken together, the brown module genes might be promising biomarkers for screen patients unsuitable for mRNA vaccination.

To further explore core genes in brown gene module, protein-protein interaction (PPI) analysis was performed by Metascape, ten hub genes were identified including Fibroblast Growth Factor 17 (*FGF17*), HRas Proto-Oncogene, GTPase (*HRAS*), Platelet Derived Growth Factor Subunit B (*PDGFB*), Kinase Insert Domain Receptor (*KDR*), Phosphoinositide-3-Kinase Regulatory Subunit 3 (*PIK3R3*), Nerve Growth Factor (*NGF*), Placental Growth Factor (*PGF*), Platelet Derived Growth Factor D (*PDGFD*), Fibroblast Growth Factor Receptor 1 (*FGFR1*), Neuropilin 1 (*NRP1*), and so on (**Figure 7F**). These findings revealed that these hub genes could be potential biomarkers for identifying suitable patients for mRNA vaccination.

## Discussion

THCA is one of the most diagnosed malignancies worldwide, and its different subtypes result in different outcomes (26). The overall prognosis of patients with differentiated thyroid cancer is favorable, but the patients with iodine-resistant differentiated thyroid cancer, distant metastatic differentiated thyroid cancer, poorly differentiated cancer, undifferentiated cancer as well as distant metastatic medullary cancer are poor due to the limited treatment options. So far, multiple strategies for cancer therapy have achieved notable progress, including targeted therapies (27), immunotherapy (28), antibody drug conjugates (29) and others. However, the specific effects of these therapies on THCA are still unclear. mRNA vaccines represent a novel immunotherapy strategy and have recently caught public attention. They can induce tumor-specific immune responses with high potency (30). Many studies have focused on improving mRNA vaccines for immune therapy. However, the development of mRNA vaccines for THCA faced major limitations.

Here, the tumor-associated antigens in THCA were identified via a comprehensive analysis of somatic mutations and amplified genes, which revealed that *TK1* could serve as a potent antigen target of THCA. *TK1* stands for thymidine kinase 1, an isoform of thymidine kinase and a cell cycle-regulated enzyme that is expressed during the early S phase and late G1 phase of the cell cycle (31). It is involved in the progression of multiple cancers, including breast cancer (32), hepatocellular carcinoma (33), esophageal squamous cell carcinoma (34), prostate cancer (35), and lung cancer (36). *TK1* was considered as a candidate agent for tumor prognosis. *TK1* overexpression was correlated to worse clinical outcomes, and *TK1* also exhibited a strong association with immune cells. Taken together, *TK1* can serve as the prognosis-related immune antigen of THCA vaccine development.

Increasing evidence highlights the limitation of patients that can benefit from vaccine-based tumor therapy (37). Thus, it is necessary to recognize suitable patients for tumor vaccines. Three novel immune subtypes were classified on the basis of the expression pattern of immune-associated genes, showing an extremely distinct prognosis and tumor immune microenvironment. The S2 subtype exhibited an immunological “cold” phenotype characterized by a lack of immune infiltration, thereby showing a non- inflammatory tumor microenvironment. S1 and S3 displayed the opposite immunologic as immunological “hot” phenotypes with abundant immune infiltration, thereby appearing an extremely inflammatory microenvironment. ICD reportedly provided a rich source of immunogens and sensitized cancer cells to interventional immunotherapy, which played essential roles in the tumor transformation from “cold” to “hot” (38). In this study, S2 was related to the high expression levels of ICDs, such as *CALR*, *EIF2A*, and *TLR3*, which suggested that S2 patients could be easily transformed into “hot” tumors and benefited from mRNA vaccine. Meanwhile, S1 and S3 patients would probably benefit from immune checkpoint inhibitors due to their high levels of ICP. Furthermore, immune infiltration analysis was performed, and we observed low infiltrations of immune cells in S2 patients, which verified that S2 could serve as a candidate population for mRNA vaccination. Therefore, patients with subtype S2 identified in this study are more likely to benefit from immunotherapy, and further immunotherapy including mRNA vaccine for patients with this subtype can be more targeted and have better therapeutic effects. It also provides a feasible method for the treatment of some types of thyroid cancer with poor prognosis.

Next, the immune landscape of THCA was constructed to recognize specific beneficial populations for tumor mRNA vaccines. Besides, the hub genes of the distinct modules were identified and analyzed, and they were mainly involved in immune-associated pathways, including MAPK pathway, neuroactive ligand-receptor interaction, cytokine-cytokine receptor interaction, and cell chemotaxis. These findings indicated the crucial role of the hub genes in the THCA microenvironment.

With the development of the sequencing, we believe that sequencing technology will be widely used in the precision medicine in the future. Through simple sequencing of cancer tissue from THCA patients, the nature of the tumor (“hot” or “cold”) will be identified. By further analysis of immunotyping we constructed, we can determine whether the patient is S2 subtype, a suitable population for the mRNA vaccine in THCA.

## Conclusions

*TK1* was identified as a promising THCA antigen for developing mRNA vaccines. Patients with immune subtype S2 are potential candidates for mRNA vaccination. Our findings provided novel insights into THCA-mRNA vaccine development, which may contribute to the precise immunotherapy of THCA.

## Data Availability

The datasets presented in this study can be found in online repositories. The names of the repository/repositories and accession number(s) can be found in the article/**Supplementary Material**.
