# Supplementary material for: Potential tumor-specific antigens and immune landscapes identification for mRNA vaccine in thyroid cancer
Source: Front Oncol. 2024 Sep 30;14:1480028. doi: 10.3389/fonc.2024.1480028 (PMC11471445; doi:10.3389/fonc.2024.1480028)
Supplement: Supplementary file 1 [file DataSheet1.docx]

**Supplementary Figure**


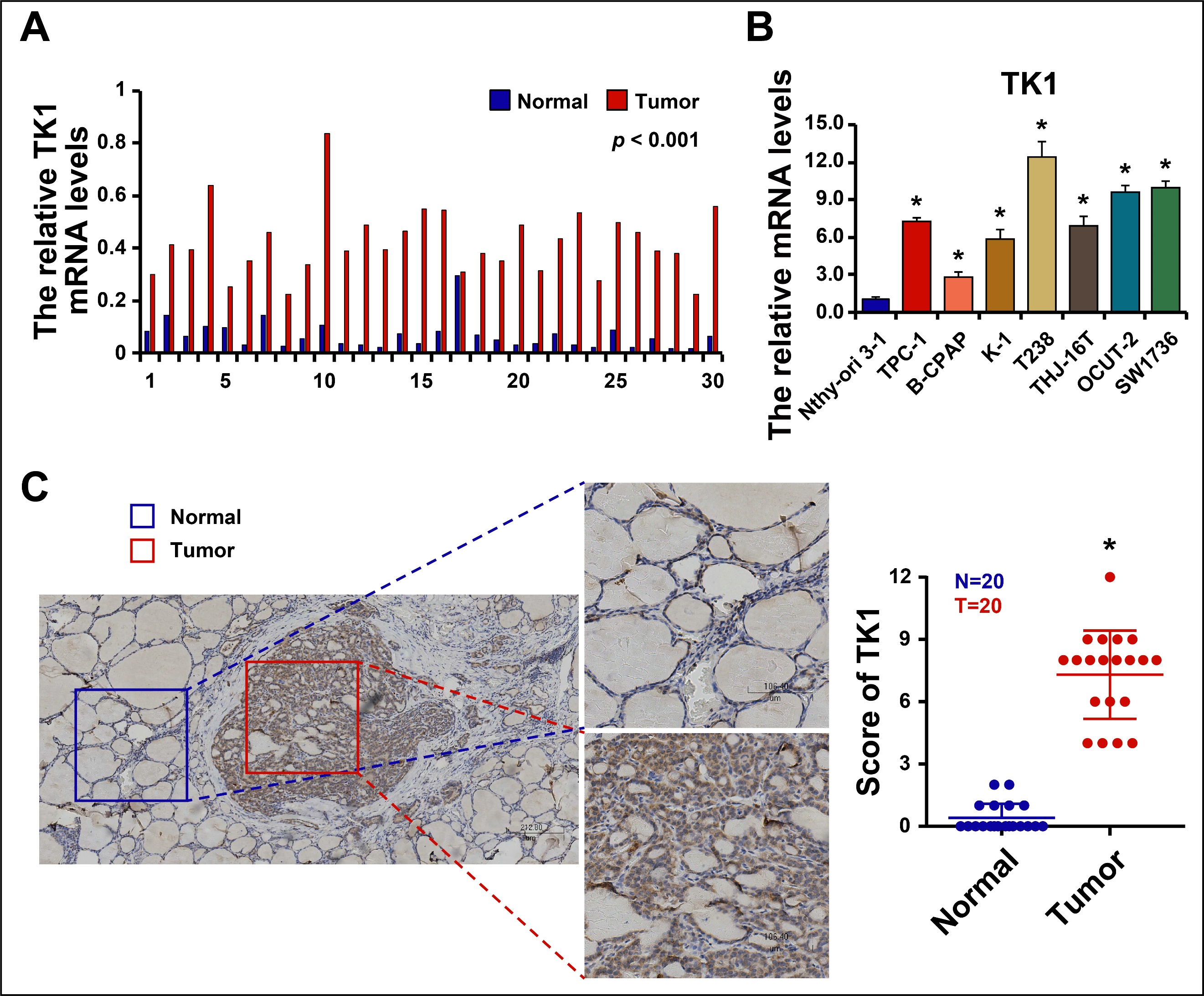


**Figure S1. The Expression Levels of TK1 in Tissues and Cell lines of Thyroid Cancer Patients.** (A) The mRNA level of TK1 in 30 paired cancer tissues and normal tissues of thyroid cancer patients detected by real-time quantitative PCR (RT-qPCR). (B) The mRNA level of TK1 in normal thyroid cells and thyroid cancer cell lines detected by RT-qPCR. (C) The protein level of TK1 from patients with thyroid cancer detected by immunohistochemistry. **P* <0.05.
